# Chronic IL-1 Exposed AR^+^ PCa Cell Lines Show Conserved Loss of IL-1 Sensitivity and Evolve Both Conserved and Unique Differential Gene Expression Profiles

**Published:** 2021-12

**Authors:** Shayna E. Thomas-Jardin, Mohammed S. Kanchwala, Haley Dahl, Vivian Liu, Rohan Ahuja, Reshma Soundharrajan, Nicole Roos, Sydney Diep, Amrit Sandhu, Chao Xing, Nikki A. Delk

**Affiliations:** 1 Biological Sciences Department, The University of Texas at Dallas, Richardson, TX 75080, USA; 2 McDermott Center of Human Growth and Development, The University of Texas Southwestern Medical Center, Dallas, TX 75390, USA; 3 Department of Bioinformatics, The University of Texas Southwestern Medical Center, Dallas, TX 75390, USA; 4 Department of Population and Data Sciences, The University of Texas Southwestern Medical Center, Dallas, TX 75390, USA

**Keywords:** Chronic inflammation, Interleukin-1, IL-1, Prostate cancer, Gene expression

## Abstract

**Introduction:**

Inflammation drives prostate cancer (PCa) progression. While inflammation is a cancer hallmark, the underlying mechanisms mediating inflammation-induced PCa are still under investigation. Interleukin-1 (IL-1) is an inflammatory cytokine that promotes cancer progression, including PCa metastasis and castration resistance. We previously found that acute IL-1 exposure represses PCa *androgen receptor* (*AR*) expression concomitant with the upregulation of pro-survival proteins, causing *de novo* accumulation of castration-resistant PCa cells. However, acute inflammation is primarily anti-tumorigenic, while chronic inflammation is pro-tumorigenic. Thus, using the LNCaP PCa cell line as model, we found that PCa cells can evolve insensitivity to chronic IL-1 exposure, restoring AR and AR activity and acquiring castration resistance. In this paper we expanded our chronic IL-1 model to include the MDA-PCa-2b PCa cell line to investigate the response to acute versus chronic IL-1 exposure and to compare the gene expression patterns that evolve in the LNCaP and MDA-PCa-2b cells chronically exposed to IL-1.

**Methods:**

We chronically exposed MDA-PCa-2b cells to IL-1α or IL-1β for several months to establish sublines. Once established, we determined subline sensitivity to exogenous IL-1 using cell viability assay, RT-qPCR and western blot. RNA sequencing was performed for parental and subline cells and over representation analysis (ORA) for geneset enrichment of biological process/pathway was performed.

**Results:**

MDA-PCa-2b cells repress AR and AR activity in response to acute IL-1 exposure and evolve insensitivity to chronic IL-1 exposure. While cell biological and molecular response to acute IL-1 signaling is primarily conserved in LNCaP and MDA-PCa-2b cells, including upregulation of NF-κB signaling and downregulation of cell proliferation, the LNCaP and MDA-PCa-2b cells evolve conserved and unique molecular responses to chronic IL-1 signaling that may promote or support tumor progression.

**Conclusions:**

Our chronic IL-1 subline models can be used to identify underlying molecular mechanisms that mediate IL-1-induced PCa progression.

## Introduction

Prostate cancer (PCa) is the second leading cause of cancer-related death in American men behind lung cancer [1]. Metastatic PCa is responsible for this high burden of mortality, as the five-year survival rate drops from almost 100% for PCa confined to the prostate to 30% for metastatic disease [1]. Metastatic PCa is currently incurable, and with the annual burden of metastatic PCa expected to rise 42% by 2025 [2], the need to understand the underlying mechanisms of PCa disease initiation and progression cannot be overstated.

As part of immunosurveillance, immunes cells infiltrate the tumor microenvironment and secrete cytotoxic and cytostatic inflammatory cytokines [3,4]. Thus, acute inflammation is anti-tumorigenic, but if left unresolved, chronic inflammation ensues [3,4]. Chronic inflammation promotes cancer [3,4], including PCa initiation and progression [5]. While chronic inflammation in the tumor microenvironment is an established hallmark of cancer [6], the underlying mechanisms of inflammation-induced disease are not fully elucidated.

Interleukin-1 (IL-1) is an inflammatory cytokine secreted by tumor cells, myeloid precursor cells, macrophages, and neutrophils in the tumor microenvironment [7,8]. IL-1 induces epithelial to mesenchymal transition, metastasis and bone colonization, angiogenesis, and autocrine and paracrine secretion of pro-tumorigenic molecules such as VEGF, IL-6, and IL-8 [9–14]. As such, the FDA approved IL-1 receptor antagonist (IL-1Ra), anakinra, is in clinical trials for breast, pancreatic, and colorectal cancer to block IL-1-induced angiogenesis, metastasis, and chemotherapy resistance (clinicaltrials.gov). In PCa, IL-1 promotes castration resistance, anti-androgen resistance, neuroendocrine differentiation, metastasis, and bone colonization [8–13,15,16]. IL-1 levels are elevated in cancer patients [17], including in PCa patient serum [18] and IL-1 levels correlate with advanced Gleason score in primary PCa tumors [12]. We have shown that IL-1Ra can attenuate bone marrow stromal cell paracrine-induced IL-1 signaling in PCa cell lines *in vitro* [19]. However, we have recently shown that LNCaP PCa cells chronically exposed to IL-1 for several months evolve resistance to exogenous IL-1 and acquire pro-tumorigenic phenotypes [16] that, as a consequence, can no longer be mitigated by IL-1Ra; thus, emphasizing the need for alternative target molecules and therapies against chronic IL-1-induced tumorigenicity.

In this study, we compared our RNA sequencing and bioinformatics analysis of LNCaP and MDA-PCa-2b sublines isolated from cultures chronically exposed for several months to IL-1 to identify the conserved and divergent gene expression changes and predicted pathways that are known to promote tumorigenicity, and thus, could underlie chronic IL-1-induced PCa progression and serve as novel therapeutic targets. Both LNCaP and MDA-PCa-2b sublines evolved insensitivity to exogenous IL-1-induced cytotoxicity, cytostaticity, and intracellular signaling and show conserved basal gene expression in a relatively small subset of genes that encode for proteins implicated in cancer, including PCa. LNCaP and MDA-PCa-2b chronic IL-1 sublines share 55 basally high genes and 17 basally low genes. Many of the shared genes reportedly have functional roles in castration resistance and/or correlate with PCa disease progression. Notably, while constitutive NF-κB signaling, a canonical IL-1-induced intracellular pathway, promotes PCa castration resistance [20,21], the LNCaP and MDA-PCa-2b sublines do not show constitutive upregulation of canonical NF-κB target genes, suggesting that chronic IL-1 can drive NF-κB-independent PCa disease. Taken together, chronic IL-1 exposure selects for cytotoxic- and cytostatic-resistant cells that evolve a gene expression profile favoring disease progression.

## Materials and Methods

### Cell culture

LNCaP (ATCC, Manassas, VA; CRL-1740), MDA-PCa-2b (ATCC, Manassas, VA; CRL-2422) and subline prostate cancer (PCa) cell lines were maintained in a 37°C, 5.0% (v/v) CO_2_ growth chamber. LNCaP and LNCaP sublines, LNas1 and LNbs1, were cultured in DMEM (Gibco/Thermo Scientific; 1185–092) supplemented with 10% (v/v) fetal bovine serum essence (FBE; Seradigm; 3100–500), 0.4 mM L-glutamine (L-glut; Gibco/Invitrogen; 25030–081), and 10 U/ml penicillin G sodium and 10 mg/ml streptomycin sulfate (pen-strep; Gibco/Invitrogen; 15140–122). MDA-PCa-2b and MDA-PCa-2b sublines, MDA-αs1 and MDA-βs1, were cultured in BRFF-HPC1, (#0403, AthenaES, Baltimore, MD) supplemented with 20% (v/v) FB essence, 0.4mM L-glut, and 10 U/mL Gibco penicillin G sodium and 10 mg/mL streptomycin sulfate (pen-strep; #15140–122, Thermo Fisher Scientific).

### Chronic IL-1 subline generation and maintenance

MDA-PCa-2b cells were maintained in HPC1/20% FB Essence (FBE) containing 0.5 ng/ml IL-1α (Gold Bio, St. Louis, MO; 1110–01A-10) or IL-1β (Gold Bio, St. Louis, MO; 1110–01B-10) for approximately 4 months; and the proliferative colonies that emerged were expanded and termed MDA-PCa-2b IL-1α subline (MDA-αs) and MDA-PCa-2b IL-1β subline (MDA-βs). We generated two sets of sublines (MDA- αs1, MDA- αs2, MDA- βs1, MDA- βs2). During subline expansion, the sublines were maintained in 0.5 ng/ml IL-1 up to an additional 4 months. During subline generation and expansion, MDA-PCa-2b parental cells were cultured in vehicle control (0.1% BSA) in 1 × PBS) alongside the sublines. Similar passage number for parental and subline cells were characterized. For phenotypic characterization, established sublines are cultured in the absence of IL-1 for at least one month to ensure the sublines are stable. Cell line authentication was previously performed for LNCaP parental, LNas1, LNbs1 [16]. Cell line authentication was performed for MDA-PCa-2b parental 1, MDA-αs1 and MDA-βs1 was performed by STR profiling by the DNA Genotyping Core, UTSW Medical Center. All cell lines used in the study are commercially available and purchased from American Tissue Culture Collection (ATCC). We hereby confirm that none of the used cell lines require any ethics approval for their use.

### Cell treatments

Human recombinant IL-1α (GoldBio, St. Louis, MO; 1110–01A-100) or IL-1β (GoldBio, St. Louis, MO; 1110–01B-100) were resuspended in 0.1% bovine serum albumin (BSA) (Thermo Fisher Scientific, Fair Lawn, NJ; BP 1600–1) in 1X phosphate buffered saline (PBS; Corning, Manassas, VA; 21–040-CM). Cells were treated with vehicle control (0.1% BSA in 1X PBS) or IL-1 added to growth medium.

### RNA isolation and reverse transcription quantitative PCR (RT-QPCR):

Total RNA was extracted, reverse transcribed, and analyzed by RT-QPCR as previously described [19]. Primer sequences for genes of interest are listed below. Gene of interest cycle times (CT) were normalized to the β-actin. Relative mRNA levels were calculated using the 2-ΔΔCT method. *Primer sequences 5’–3’*: *Beta actin (β-actin)*, forward GATGAGATTGGCATGGCT TT, reverse CACCTTCACCGGTCCAGTTT; *Mitochondrial Superoxide Dismutase 2 (SOD2)*, forward GGCCTACGTGAACAACCTGA, reverse GTTCTCCACCACCGTTAGGG; *Lipocalin-2 (LCN2*), forward TCACCCTCTACGGGAGAACC, reverse GGGACAGGGA AGACGATGTG; *NADPH Oxidase 1 (NOX1)*, forward TGGTTGT TTGGTTAGGGCTGAAT, reverse AGTGTGCGGCTGCAAAACC; *Interleukin-1 Receptor Type 1*(*IL-1R1*), forward TGGGGAAGG GTCTACCTCTG, reverse TCCCCAACGTAGTCATCCCT; *Follistatin Like 1 (FSTL1*), forward CAACCACTGTGAACTGCATCG, reverse GATAGCAAACAACTGGGCTGG; *T-cell receptor gamma alternate reading frame protein (TARP)*, forward GCTGAA ACAAAGCTCCAGAAGG, reverse TCCCAGAATCGTGTTGCTCT; *Cancer/Testis Antigen 2 (CTAG2),* forward ACATCACGATGCCTT TC TCGTC, reverse AGCAGTCAGTCGGATAAACAGT; *Prostaglandin Dehydrogenase 1 (HPGD),* forward GCTTAAGGGCGCCAAGGTA, reverse CCACATCGCACTGGATGAAC.

### RNA-sequencing (RNA-seq) analysis

LNCaP cells treated with vehicle control or 25 ng/ml IL-1β [19] (GSE105088) and untreated LNCaP parental, LNas1 and LNbs1[16] (GSE142706) were previously sequenced. RNA-seq was performed for MDA-PCa-2b parental 1 cells treated with vehicle control (0.1% BSA in 1xPBS) or 25 ng/ml IL-1α or IL-1β for 3 days and MDA-αs1 and MDA-βs1 grown in vehicle control for 3 days (GSE181229). RNA-seq was performed by the Genome Center at the University of Texas at Dallas (Richardson, TX). Total RNA library was prepared using Illumina Truseq Stranded Total RNA prep Gold kit (Illumina). The prepared libraries were sequenced on an Illumina NextSeq 500 platform (San Diego, CA) with 75bp single-end reads. Fastq files were checked for quality using fastqc (v0.11.2) [22] and fastq_screen (v0.4.4) [23] and were then mapped to hg19 (UCSC version from igenomes) using STAR [24]. Read counts were generated using feature Counts [25] and differential expression analysis was performed using edgeR [26,27]. Differential gene expression lists were generated using the following cut-offs: log2 counts per million (CPM) ≥ 0, log2 fold change (FC) ≥ 0.6 or ≤ −0.6, false discovery rate (FDR) ≤ 0.05, adjusted p-value ≤ 0.05. Pathway analysis was conducted using QIAGEN’s Ingenuity Pathway Analysis (IPA) tool (http://www.qiagen.com/ingenuity) RNA-seq datasets generated for this study are available at GEO NCBI, accession GSE181229.

### Over representation analysis

Over representation analysis (ORA) was performed using the WEB-based Gene SeT AnaLysis Toolkit (WebGestalt) site (http://www.webgestalt.org/) [28]. Gene lists were analyzed using the geneontology biological process functional database. The parameters for the enrichment analysis are minimum/maximum genes in a category = 5/2000, FDR method = BH, number of categories to report = top 40. Induced and repressed gene lists were uploaded separately to identify, respectively, upregulated and downregulated biological processes.

### Western blot

Protein was isolated from cells using NP40 lysis buffer (0.5% NP40 [US Biological, Salem, MA; N3500], 50 mM of Tris [pH 7.5], 150 mM of NaCl, 3 mM of MgCl2, 1X protease inhibitors [Roche, Mannheim, Germany; 05892953001]). Protein concentration was measured using the Pierce BCA Protein Assay Kit (Thermo Fisher Scientific, Waltham, MA; 23227). For Western blot analysis, equal protein concentrations were loaded onto and separated in 12% (wt/vol) sodium dodecyl sulfate polyacrylamide gel (40% acrylamide/bisacrylamide solution; Bio-Rad, Hercules, CA; 161–0148). Proteins were transferred from the gel to 0.45 μm pore size nitrocellulose membrane (Maine Manufacturing, Sanford, ME; 1215471) and total protein visualized using Ponceau S (Amresco, Radnor, PA; K793). The membrane was blocked with 2.5% (wt/vol) BSA (Thermo Fisher Scientific, Waltham, MA; BP 1600–1) in 1X tris-buffered saline with Tween 20 (TBST; 20 mM of Tris, pH 7.6, 150 mM of NaCl, 0.05% Tween-20). Primary and secondary antibodies were diluted in 2.5% BSA in 1X TBST. Protein blot bands were visualized using Clarity Western ECL Substrate (Bio-Rad, Hercules, CA; 1705061) or SuperSignal West Femto Maximum Sensitivity Substrate (Thermo Scientific, Rockford, IL; 34095) and imaged using Amersham Imager 600 (GE, Marlborough, MA). *Primary antibodies:* AR (Cell Signaling, Danvers, MA; D6F11), β-actin (Santa Cruz, Santa Cruz, CA; sc-69879), LCN2 (Cell Signaling, Danvers, MA; D4M8L), NKX3.1(Cell Signaling, D2Y1A), PARP (Cell Signaling, Danvers, MA; 9532S), PSA (Cell Signaling, Danvers, MA; 5365S), SOD2 (Abgent, San Diego, CA; AM7579a). *Secondary antibodies:* Sheep anti-mouse (Jackson ImmunoResearch Laboratories; 515-035-062), goat anti-rabbit (Abnova; PAB10822).

### MTT (3-(4,5-Dimethylthiazol-2-yl)-2,5-diphenyltetrazolium bromide) viability assay

MTT assay (Trevigen, Gaithersburg, MD; 4890–25-K) was performed according to manufacturer’s instructions. Cell viability was quantified as the optical density (OD) read at wavelengths 540 nm and 650 nm. The final OD was calculated as follows: OD 540 nm – OD 650 nm. OD was measured using the Cytation3 Imaging Reader (BioTek, Winooski, VT).

### Statistical analysis

Statistical significance for MTT and cell counts was determined using unpaired student t-test calculated using Microsoft Excel. P-values of ≤ 0.05 were considered statistically significant and denoted by asterisks (*p ≤ 0.05; **p ≤ 0.05; ***p ≤ 0.005). Graphs are shown as the average of a minimum of n = 3 biological replicates ± standard deviation (STDEV).

## Results

### MDA-PCa-2b cells evolve insensitivity to chronic IL-1 exposure

**Figure 1:**
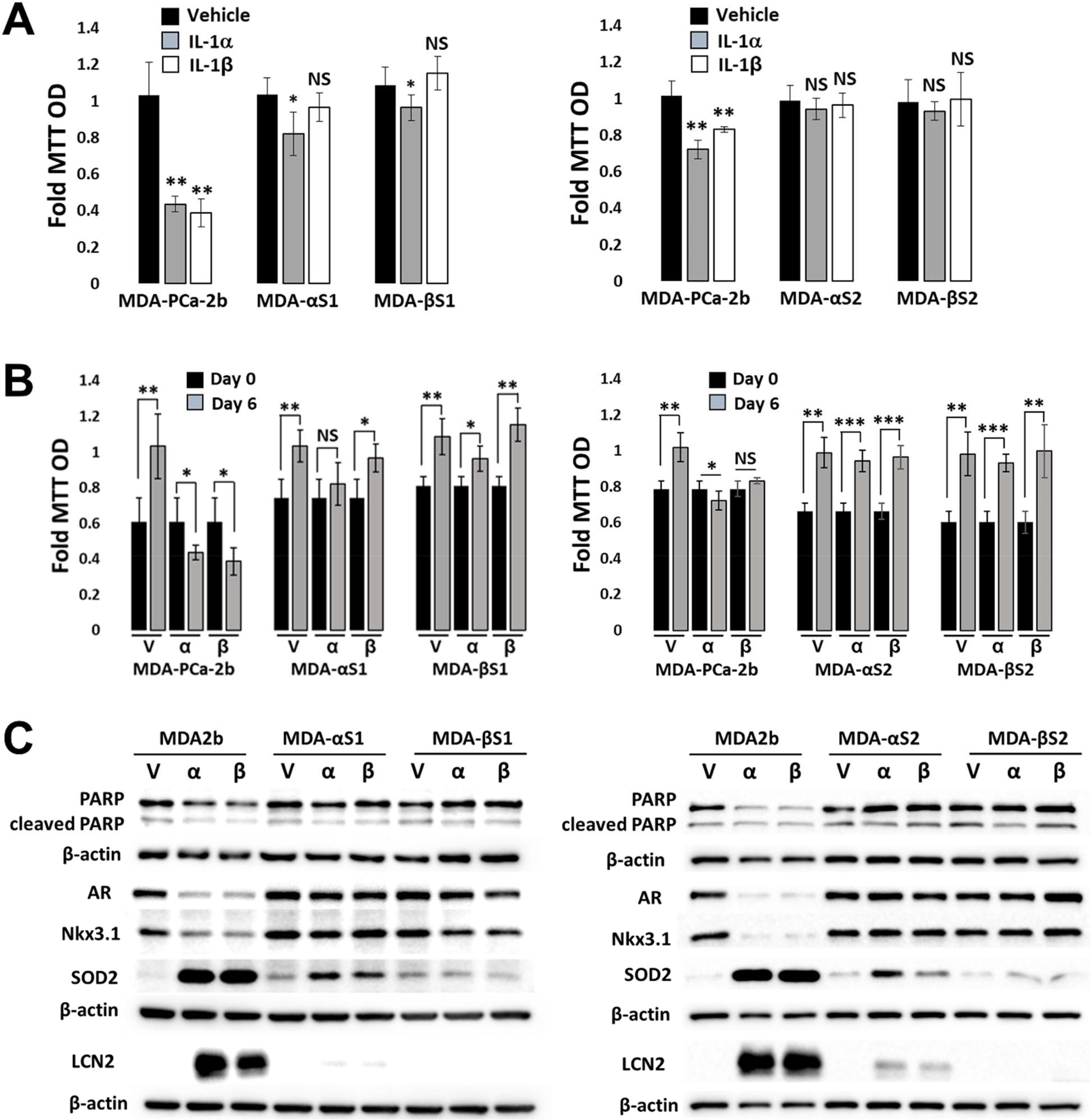
MDA-PCa-2b chronic IL-1 sublines evolve insensitivity to IL-1-induced cytotoxicity, cytostaticity, and intracellular signaling. Parental MDA-PCa-2b (MDA2b) and chronic IL-1 subline cells (MDA-αs1, MDA-αs2, MDA-βs1, MDA-βs2) were treated acutely for 3 days (A, C) or 6 days (B) with vehicle control (V), 25 ng/ml IL-1α (a), or 25 ng/ml IL-1β (b) and analyzed for cell viability using MTT (A, B) or protein accumulation by western blot (C). Acute IL-1 exposure reduces cell viability and proliferation, reduces full-length PARP (indicative of cell death activation), induces SOD2 and LCN2 protein accumulation (canonical IL-1-induced genes), and reduces AR and NKX3.1 (canonical AR target gene) protein accumulation in MDA-PCa-2b parental cells, but has little to no effect on the chronic IL-1 sublines. Thus, the IL-1 sublines evolved insensitivity to IL-1. Error bars, ± STDEV of 4 biological replicates; p-value, *≤ 0.05, **≤ 0.005, ***≤ 0.005, NS = not significant. Fold MTT optical density (OD) is normalized to treatment control. β-actin is the western blot loading control.

To investigate the effect of chronic IL-1 exposure on MDA-PCa-2b cells, cells were cultured in 0.5 ng/ml IL-1α or IL-1β for 3–4 months, as previously described for LNCaP cells [16]. IL-1α and IL-1β (hereinafter, collectively referred to as IL-1) are the two main IL-1 cytokine family members, both bind the IL-1 receptor and have similar biological activity [7]. As with LNCaP cells [16,29], IL-1 is cytotoxic (Figure 1A) and cytostatic (Figure 1B) for MDA-PCa-2b cells; but after 3–4 months cultured in IL-1, proliferative colonies emerged and were expanded to generate sublines. For rigor, we generated two sets of MDA-PCa-2b chronic IL-1 sublines that we termed MDA-PCa-2b IL-1α subline 1 (MDA-αs1), MDA-PCa-2b IL-1α subline 2 (MDA-αs2), MDA-PCa-2b IL-1β subline 1 (MDA-βs1), and MDA-PCa-2b IL-1β subline 2 (MDA-βs2).

**Figure 2:**
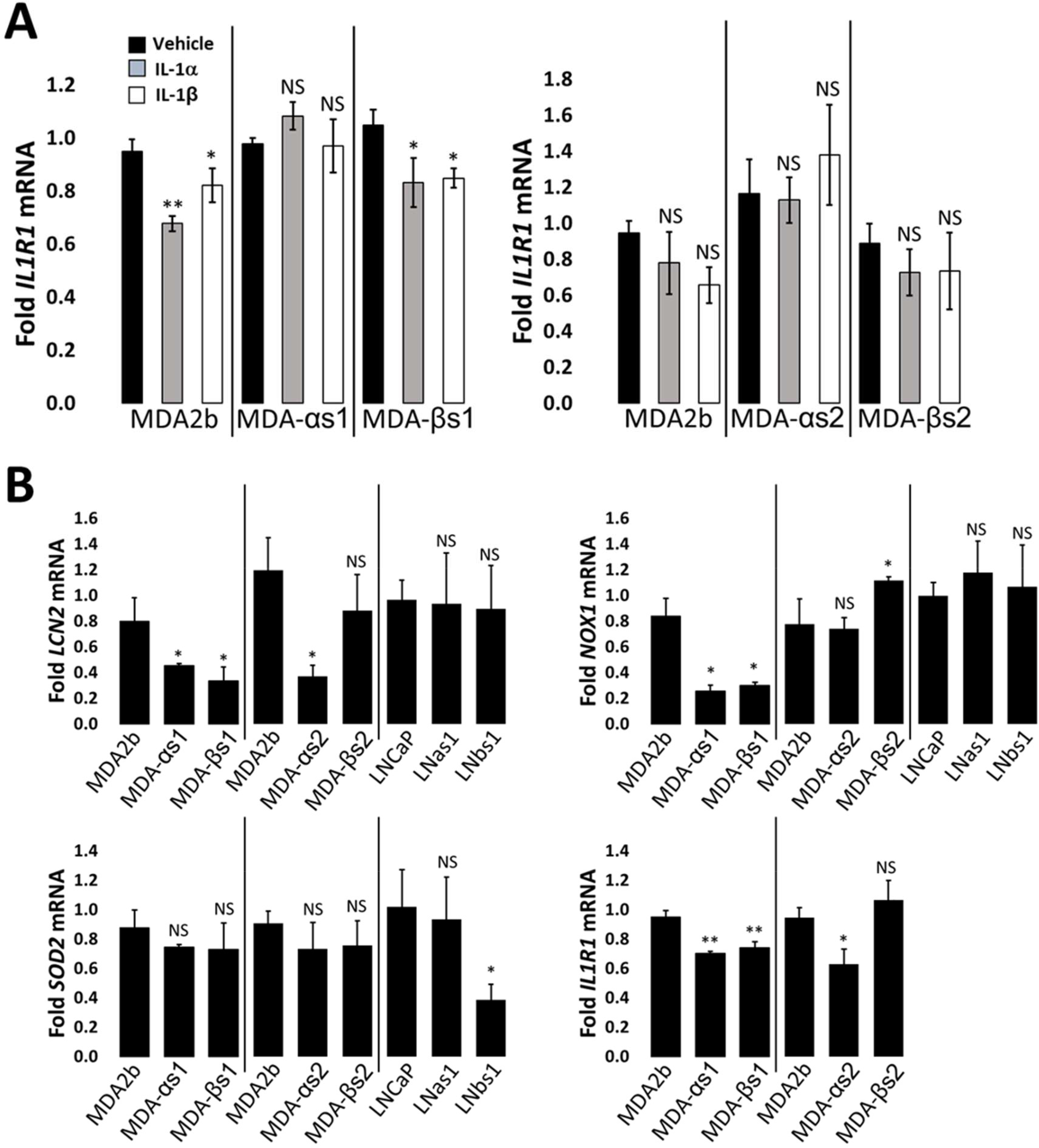
Chronic IL-1 MDA-PCa-2b and LNCaP cell lines do not evolve constitutive NF-κB signaling. (A) MDA-PCA-2b parental (MDA2b) and chronic IL-1 subline (MDA-αs1, MDA-αs2, MDA-βs1, MDA-βs2) cells were treated acutely for 3 days with vehicle control, 25 ng/ml IL-1α, or 25 ng/ml IL-1β and analyzed by RT-qPCR for mRNA levels of the IL-1 receptor, *IL-1R1*. Acute IL-1 exposure does not increase IL-1 receptor (*IL-1R1*) mRNA levels in parental cells, suggesting basal *IL-1R1* levels are sufficient to mediate IL-1 signaling. Furthermore, IL-1 does not show a differential effect on *IL-1R1* mRNA levels in parental versus subline cells, suggesting subline insensitivity is independent of IL-1R1 levels. (B) Vehicle control treated cells were compared for basal mRNA levels of IL-1R1 and of canonical IL-1-induced genes, *LCN2*, *NOX1*, and *SOD2*. *IL-1R1, LCN2*, *NOX1*, and *SOD2* basal mRNA levels are comparable across the parental and subline cells, suggesting chronic IL-1 exposure does not induce constitutive activation of canonical IL-1 intracellular signaling. These data suggest that MDA-PCa-2b cell lines evolve insensitivity to exogenous chronic IL-1 exposure independent of *IL-1R1* levels or constitutive activation of intracellular IL-1 signaling. Error bars, ± STDEV of 3 biological replicates; p-value, *≤ 0.05, **≤ 0.005, ***≤ 0.005, NS = not significant. For IL-1-treated cells, mRNA levels are normalized to vehicle control for each cell line. For basal expression, mRNA levels are normalized to the parental cell line.

To demonstrate that the MDA-PCa-2b chronic IL-1 sublines acquired insensitivity to IL-1, the parental and subline cells were given an acute IL-1 exposure of 25 ng/ml IL-1 for 3 or 6 days. Acute IL-1 exposure reduced cell viability (Figure 1A) and proliferation (Figure 1B), reduced full-length PARP (indicative of cell death activation), and upregulated the protein accumulation of the canonical IL-1-induced genes, *Super Oxide Dismutase* (*SOD2*) and *Lipocalin 2* (*LCN2*) (Figure 1C). Acute IL-1 also represses AR levels and activity in AR^+^ PCa cell lines [19,30]. As such, acute IL-1 treatment repressed AR and NKX3.1 (canonical AR-induced gene) protein accumulation in the parental cells, but not in the subline cells (Figure 1C). Finally, while acute IL-1 exposure represses AR accumulation and activity in MDA-PCa-2b parental cells, as observed for LNCaP [16], the chronically exposed subline cells have restored AR accumulation and activity (Figure 1C). Taken together, these results demonstrate that chronic IL-1 exposure selects for MDA-PCa-2b cells that evolve insensitivity to exogenous IL-1. Of note, mRNA levels of the IL-1 receptor, IL-1R1, are not induced by IL-1 in parental cells (Figure 2A), indicating that basal IL-1R1 levels are sufficient to mediate IL-1 intracellular signaling in parental cells. Furthermore, IL-1 does not show a differential effect on *IL-1R1* mRNA levels in parental versus subline cells (Figure 2A) and *IL-1R1* mRNA levels are comparable in parental versus subline cells (Figure 2B), suggesting subline insensitivity to exogenous IL-1 is independent of IL-1R1 levels.

### Over representation analysis (ORA) of differentially expressed genes predicts acute and chronic IL-1 response in the LNCaP and MDA-PCa-2b cells

**Figure 3:**
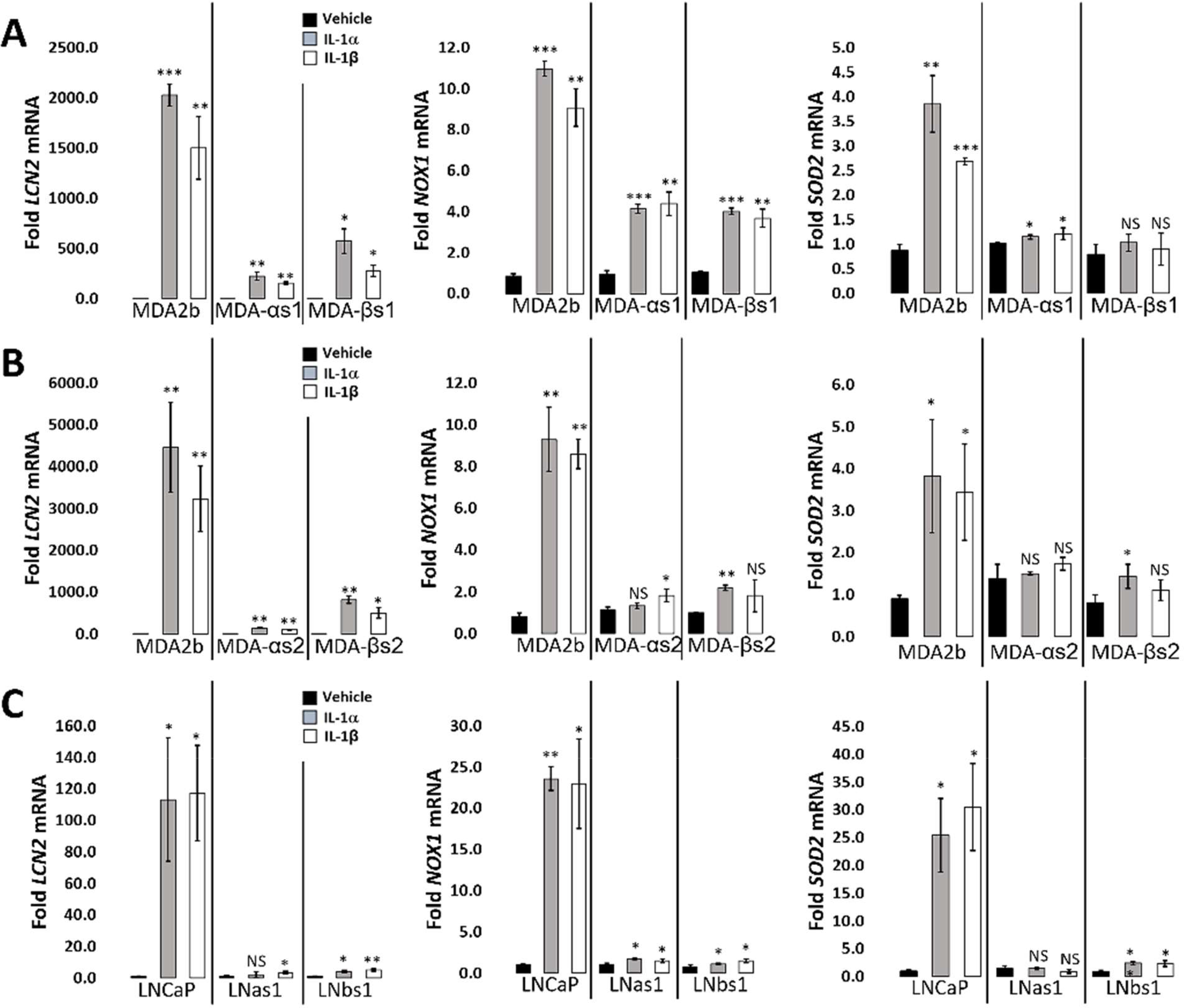
Conservedacute and chronic IL-1 response in MDA-PCa-2b and LNCaP cell lines. Parental (MDA-PCA-2b (MDA2b), LNCaP) and subline (MDA-αs1, MDA-αs2, MDA-βs1, MDA-βs2, LNas1, LNbs1) cells were treated acutely for 3 days with vehicle control, 25 ng/ml IL-1α, or 25 ng/ml IL-1β and analyzed by RT-qPCR for mRNA levels (A, B, C). Acute IL-1 exposure increases *LCN2*, *NOX1*, and *SOD2* mRNA levels in parental MDA-PCa-2b and LNCaP cells, but acute IL-1 exposure has attenuated or no effect on mRNA levels in the subline cells. Thus, both LNCaP and MDA-PCa-2b cell lines show conserved intracellular response to acute IL-1-induced changes mRNA levels and evolve chronic IL-1 insensitivity independent of constitutive canonical IL-1 intracellular signaling. Error bars, ± STDEV of 3 biological replicates; p-value, *≤ 0.05, **≤ 0.005, ***≤ 0.005, NS = not significant. For IL-1-treated cells, mRNA levels are normalized to vehicle control for each cell line.

RNA sequencing was previously performed for LNCaP cells treated acutely for 3 days with 25 ng/ml IL-1β [19] (GSE105088) and, here, we performed RNA sequencing for MDA-PCa-2b cells treated acutely for 3 days with 25 ng/ml IL-1α or IL-1β (GSE181229). LNCaP and MDA-PCa-2b share 927 genes upregulated by acute IL-1 exposure and share 747 repressed genes (Supplementary Table 1; Supplementary Figure 1A). We performed gene set ORA against the Gene Ontology biological process database using WebGestalt functional enrichment analysis web tool [28]. IL-1 is a cytotoxic and cytostatic inflammatory cytokine that signals through NF-κB [16,29,31,32]; thus, as expected, ORA of the shared upregulated genes predicts that cytokine and immune response pathways, including NF-κB signaling, are activated by acute IL-1 exposure in LNCaP and MDA-PCa-2b cells and ORA of the shared repressed genes predicts down regulation of the cell cycle and DNA, RNA, and protein biosynthesis (Supplementary Table 1; Supplemental Figure 1B). The ORA results are in line with our experimental data showing the LNCaP and MDA-PCa-2b cells upregulate IL-1-NF-κB induced genes [33–37] *LCN2*, *NOX1*, and *SOD2* (Figure 3) and show reduced viability and proliferation in response to IL-1 (Figure 1).

We also previously performed RNA sequencing for basal gene expression in the LNCaP chronic IL-1 sublines [16] (GSE142706) and, here, we performed RNA sequencing for basal gene expression in the MDA-PCa-2b chronic IL-1 sublines (GSE181229). We performed ORA of the suite of basally high genes in LNCaP chronic IL-1 sublines and in MDA-PCa-2b chronic IL-1 sublines to predict upregulated biological processes (Supplementary Table 1, Tab 7 and Tab 10). In line with our experimental data showing that the LNCaP and MDA-PCa-2b chronic IL-1 sublines do not have constitutively elevated mRNA levels of NFkB-induced genes (Figure 2B), NF-κB signaling is not represented in the biological pathways predicted by ORA of the high basal gene suites (Supplementary Table 1, Tab 7 and Tab 10). ORA of basally low genes in LNCaP chronic IL-1 sublines or in MDA-PCa-2b chronic IL-1 sublines predicts that inflammatory responses are repressed, as well as the negative regulation of cell proliferation and growth (Supplementary Table 1, Tab 8 and Tab 11). Taken together, our ORA and experimental results indicate the LNCaP and MDA-PCa-2b parental cells show conserved response to acute IL-1, activating IL-1-NF-κB intracellular signaling and showing cytostatic and cytotoxic cell responses. Furthermore, following chronic IL-1 exposure, both LNCaP and MDA-PCa-2b cells lose canonical IL-1-NF-κB signaling and negative regulation of cell proliferation and growth.

### Conserved and unique gene expression patterns in LNCaP and MDA-PCa-2b chronic IL-1 sublines

**Figure 4:**
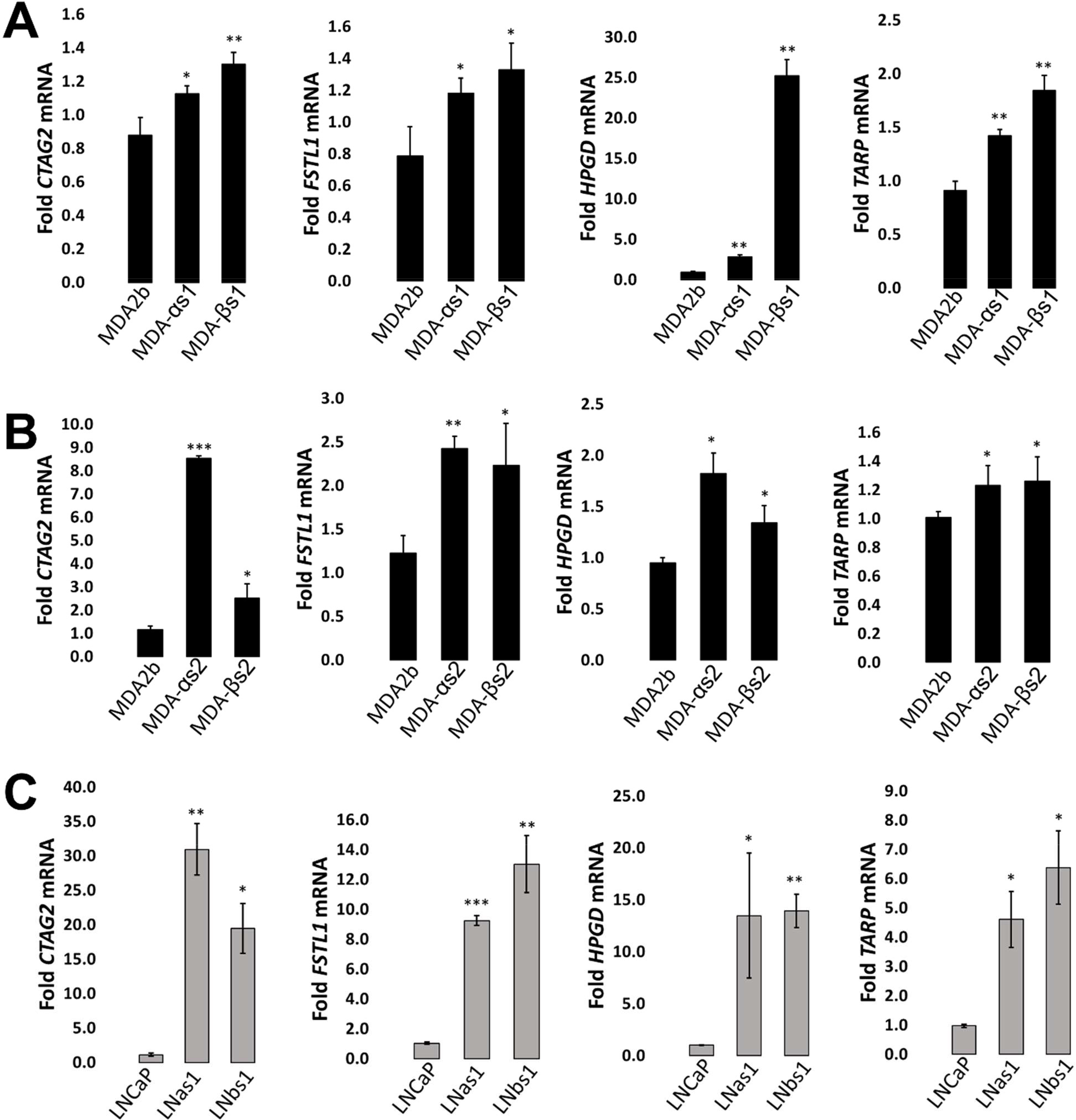
RT-qPCR confirmation of basally high expression for select genes identified by RNA sequencing. Parental (MDA-PCA-2b (MDA2b), LNCaP) and subline (MDA-αs1, MDA-αs2, MDA-βs1, MDA-βs2, LNas1, LNbs1) cells were analyzed for basal mRNA levels of *CTAG2*, *FSTL1*, *HPGD*, and *TARP* by RT-qPCR (A, B, C). *CTAG2*, *FSTL1*, *HPGD*, and *TARP* are basally high in the chronic IL-1 sublines relative to parental cells. Error bars, ± STDEV of 3 biological replicates; p-value, *≤ 0.05, **≤ 0.005, ***≤ 0.005, NS = not significant. mRNA levels are normalized to the parental cell line.

Given that IL-1 promotes cancer progression [17], we compared the basal differential gene expression of LNCaP [16] and MDA-PCa-2b chronic IL-1 subline cells (Supplementary Table 1) to identify predicted tumorigenic pathways. Compared to LNCaP parental cells, there are 2954 basally high genes in LNas1 and LNbs1 chronic IL-1 sublines and 629 basally low genes (Supplementary Table 1; Supplementary Figure 2A). Compared to MDA-PCa-2b parental cells, there are 204 basally high genes in MDA-αs1 and MDA-βs1 chronic IL-1 sublines and 184 basally low genes (Supplementary Table 1; Supplementary Figure 2A). Of the differentially expressed genes in each subline set, the LNCaP and MDA-PCa-2b chronic IL-1 sublines share expression patterns in a select subset of genes. LNas1, LNbs1, MDA-αs1, and MDA-βs1 sublines share 55 basally high genes and 17 basally low genes (Supplementary Table 1; Supplementary Figure 2A). We confirmed expression of several basally high genes in the LNCaP and MDA-PCa-2b chronic IL-1 sublines that are also associated with PCa progression, *CTAG2*, *FSTL1*, *HPGD* and *TARP* (Figure 4) [38–45]. Of note, among the independently generated MDA-PCa-2b chronic IL-1 sublines, the basal mRNA levels of the select confirmed genes is variable. We believe this is due to clonal heterogeneity and genetic drift during IL-1 chronic subculturing. Nevertheless, we see conserved basally high mRNA levels for the selected genes which we will functionally investigate in future studies.

We performed ORA for the 55 basally high and 17 basally low genes which predicted several constitutively upregulated or repressed pathways in both the LNCaP and MDA-PCa-2b chronic IL-1 sublines (Supplementary Table 1; Supplementary Figure 2B). For example, ORA of the basally high gene set predicts that the chronic IL-1 sublines evolved constitutive expression of genes that regulate insulin signaling and control glucose homeostasis and ORA of the basally low gene set predicts that the chronic IL-1 sublines evolved constitutive repression of genes that regulate abiotic stress response (Supplementary Table 1; Supplementary Figure 2). Notably, the genes involved in regulating insulin signaling and glucose homeostasis, *DPP4*, *FKBP1B*, *ICAM1*, *IGF1*, *IGF1R*, *INHBB*, *PTPRN2*, *STXBP5L*, and *VWA2*, and the genes that regulate abiotic stress response, *HPN*, *DNAJB1*, *FOS*, *IER5*, *HSPA6*, and *ARNT2* (Supplementary Table 1, Tab 13 and Tab 14), are also correlated with and/or promote anti- or pro-tumorigenic phenotypes, including in PCa.

In addition to the shared gene expression pattern, the LNCaP and MDA-PCa-2b chronic IL-1 sublines show cell line-unique changes in gene expression that were acquired in response to chronic IL-1 exposure (Supplementary Table 1). We performed ORA for basal gene expression in LNas1 and LNbs1 (Supplementary Table 1, Tab 7 and Tab 8) and performed ORA for basal gene expression in MDA-αs1 and MDA-βs1 (Supplementary Table 1, Tab 10 and Tab 11). As expected, ORA predicts that LNCaP and MDA-PCa-2b chronic IL-1 sublines evolved distinct biological processes. For example, ORA predicts that cell projection and neurogenesis biological processes are upregulated and cell adhesion is downregulated in the LNas1 and LNbs1 sublines (Supplementary Table 1); while in the MDA-as1 and MDA-bs1 sublines, interferon gamma signaling is predicted to be activated and VEGF signaling is predicted to be downregulated (Supplementary Table 1). Thus, while LNCaP and MDA-PCa-2b chronic IL-1 sublines share conserved insensitivity to IL-1-induced intracellular signaling [16] (Figures 1 and 3), the broader response to chronic IL-1 exposure is unique among the cell lines. The divergent response to chronic IL-1 likely reflects genetic differences in LNCaP and MDA-PCa-2b cell lines, as could be expected for tumor cell response to chronic inflammation among different patients. Taken together, our models reveal potential therapeutic targets for individuals whose PCa disease progression evolves from chronic inflammation.

## Discussion

We have developed novel models to explore the effect of chronic inflammation on PCa progression. Specifically, we generated LNCaP [16] and MDA-PCa-2b sublines from cultures chronically exposed to IL-1 for several months. Given that IL-1 is cytotoxic and cytostatic [16,29] (Figure 1), chronic IL-1 exposure selected for viable, proliferative PCa cells that evolved IL-1 insensitivity. Interestingly, we find that castration-resistant cell lines have reduced or no sensitivity to IL-1-induced cytotoxicity and cytostaticity [16,29] (data not shown) and we and other labs have found that chronic IL-1 exposure can select for castration-resistant PCa cells [15,16]. Chronic IL-1 sublines can also develop resistance to other cytotoxic and cytostatic inflammatory cytokines [16]. Therefore, we speculate that chronic IL-1 exposure in the PCa tumor microenvironment imposes selective pressure on tumor cells to acquire constitutive pro-survival mechanisms that provide a growth advantage against cellular stresses, such as castration or acute cytostatic and cytotoxic inflammation. Thus, we can use our subline models to elucidate strategies that PCa cells elicit to escape inflammatory cytokine toxicity and growth inhibition.

Canonical IL-1 signaling occurs through the NF-κB transcription factor [31] and constitutive NF-κB activity induces PCa castration resistance [20,21]. Therefore, interventions targeting IL-1/IL-1R1 binding [17] or inhibiting NF-κB intracellular pathway activation [46] could mitigate NF-κB-dependent PCa castration resistance. But for cells that evolve resistance to IL-1 due to chronic IL-1 exposure and, likewise, do not acquire constitutive NF-κB signaling, IL-1-NF-κB-targeting strategies would be ineffective. Therefore, our chronic IL-1 cell line models can be used to identify alternative therapeutic targets.

While both LNCaP and MDA-PCa-2b cell lines evolve insensitivity to chronic IL-1 exposure, the underlying mechanism of insensitivity remains unknown. LNCaP chronic IL-1 sublines also develop insensitivity to TNFα [16] which also signals through NF-κB[31], suggesting that the resistance mechanism is downstream of the IL-1/IL-1R1 receptor/ligand interaction in LNCaP cells. *IL-1R1* mRNA levels are comparable in LNCaP parental and sublines cells [16] and comparable in MDA-PCa-2b parental and subline cells (Figure 2), implying that the IL-1 resistance mechanism is independent of IL-1R1 levels. However, it will be important to determine efficacy of the IL-1/IL-1R1 protein-protein interaction in MDA-PCa-2b cells and given the different genetic backgrounds of LNCaP and MDA-PCa-2b cell lines, it is possible that the mechanism(s) governing IL-1 insensitivity in the LNCaP versus MDA-PCa-2b chronic IL-1 sublines are not conserved. We will use our subline models to determine how cells can acquire resistance to chronic IL-1 exposure. Uncovering the mechanisms may help mitigate chronic IL-1-induced castration resistance or PCa progression.

The LNCaP and MDA-PCa-2b chronic IL-1 sublines share a select basal gene expression profile of 55 basally high and 17 basally low genes (Supplementary Table 1). Significantly, many of these genes have reported roles in cancer and may be critical for inflammation-induced PCa progression. For example, the shared basally high genes that we confirmed by RT-qPCR, *CTAG2*, *FSTL1*, *HPGD* and *TARP* are pro-tumorigenic. *CTAG2* (cancer-testis antigen-2) encodes for the autoimmunogenic tumor antigen, LAGE-1, expressed by cancer cells, including PCa [47]. Functionally, CTAG2 was shown to promote breast cancer cell invasion [48]. Phase 1 clinical trial results are pending for a combined NY-ESO-1/LAGE-1 vaccine for patients with androgen-independent metastatic PCa (NCT00616291). Metastatic castration resistant PCa (mCRPC) patients give an NY-ESO-1 (CTAG1) peptide vaccine in a Phase 1 clinical trial showed slowed PSA doubling time [38], thus, the combined NY-ESO-1/LAGE-1 vaccine is expected to be efficacious. *FSTL1* (Follistatin-like protein 1) is a proinflammatory glycoprotein [49] that has been shown to promote invasion and metastasis in colorectal cancer [50] and chemoresistance and stemness in breast cancer [51]. *FSTL1* is an AR-induced gene and is elevated in CRPC [39]. *HPGD* (15-Hydroxyprostaglandin dehydrogenase) is an anti-inflammatory enzyme that catabolizes prostaglandins [52]. *HPGD* is an AR-induced gene [40] and is elevated in primary and metastatic PCa tissue [41]. *HPGD* siRNA silencing reduced LNCaP cell viability *in vitro* [41]. *TARP* (T-cell receptor gamma alternate reading frame protein) is an autoimmunogenic tumor antigen expressed by PCa cells [42]. *TARP* is an AR-induced gene [43] and *TARP* overexpression in the PC3 PCa cell line reduced *IL-1b* expression and enhanced cell proliferation [44]. A Phase 1 clinical trial showed that TARP peptide vaccination slows PSA doubling time and tumor growth rate in Stage D0 PCa patients with biochemical PSA recurrence (NCT00972309) [45]. Taken together, the conserved molecular response to chronic IL-1 exposure may have functional significance in PCa tumorigenicity which we can elucidate using our chronic IL-1 cell line models.

Indeed, the individual genes modulated by chronic IL-1 exposure that reportedly function in PCa disease can be explored as novel therapeutic targets, but it will also be important to interrogate the signaling networks that integrate the global changes in chronic IL-1-induced gene expression, including those that are unique to the different cell backgrounds. Our ORA bioinformatic analysis provides putative tumor promoting pathways to investigate that would be acquired during chronic IL-1 exposure, such as insulin and interferon gamma signaling, glucose homeostasis and metabolism and cell adhesion.

The basal changes in gene expression in parental versus chronic IL-1 subline cells suggests that chronic IL-1 exposure drives constitutive genetic or epigenetic changes over time. LNCaP and MDA-PCa-2b intracellular signaling, gene expression patterns, ORA-predicted cell biological processes, and cell behavior in response to acute IL-1 exposure are conserved, but the gene expression patterns and ORA-predicted cell biological processes diverge as the different cell lines evolve insensitivity to chronic IL-1 signaling. This suggests that genetic and epigenetic reprogramming in response to chronic IL-1 exposure would evolve differently in different patients. Therefore, we can begin to uncover the various ways in which chronic IL-1 exposure remodels chromatin using our chronic IL-1 subline models in the different cell line backgrounds. Studies are underway to elucidate chronic IL-1-induced chromatin remodeling, the subsequent constitutive changes in signaling networks, and the contribution of these altered signaling networks to PCa progression.

As stated, while LNCaP and MDA-PCa-2b cell lines both develop insensitivity to chronic IL-1 exposure, the two cell line backgrounds evolved primarily disparate changes in gene expression, likely due to underlying genetic differences in the cell line backgrounds. Notably, however, chronic exposure to IL-1α or IL-1β both cause similar gene expression changes within a cell line background, indicating that a conserved mechanism leads to chronic IL-1 insensitivity and changes in intracellular signaling within the cell line. Therefore, we can identify master regulators of the chronic IL-1 molecular response downstream of the IL-1 ligand/receptor interaction using our subline models.

Although the LNCaP and MDA-PCa-2b chronic IL-1 sublines have lost sensitivity to IL-1-induced intracellular signaling, the sublines are still sensitive to other stimuli such as IL-6 or serum starvation (data not shown). Thus, the context of the tumor microenvironment and conventional therapy will be very important in determining the cell biological and physiological consequence of IL-1-insensitive PCa cell subpopulations in PCa progression. Those studies are underway.

## Conclusion

**Figure 5:**
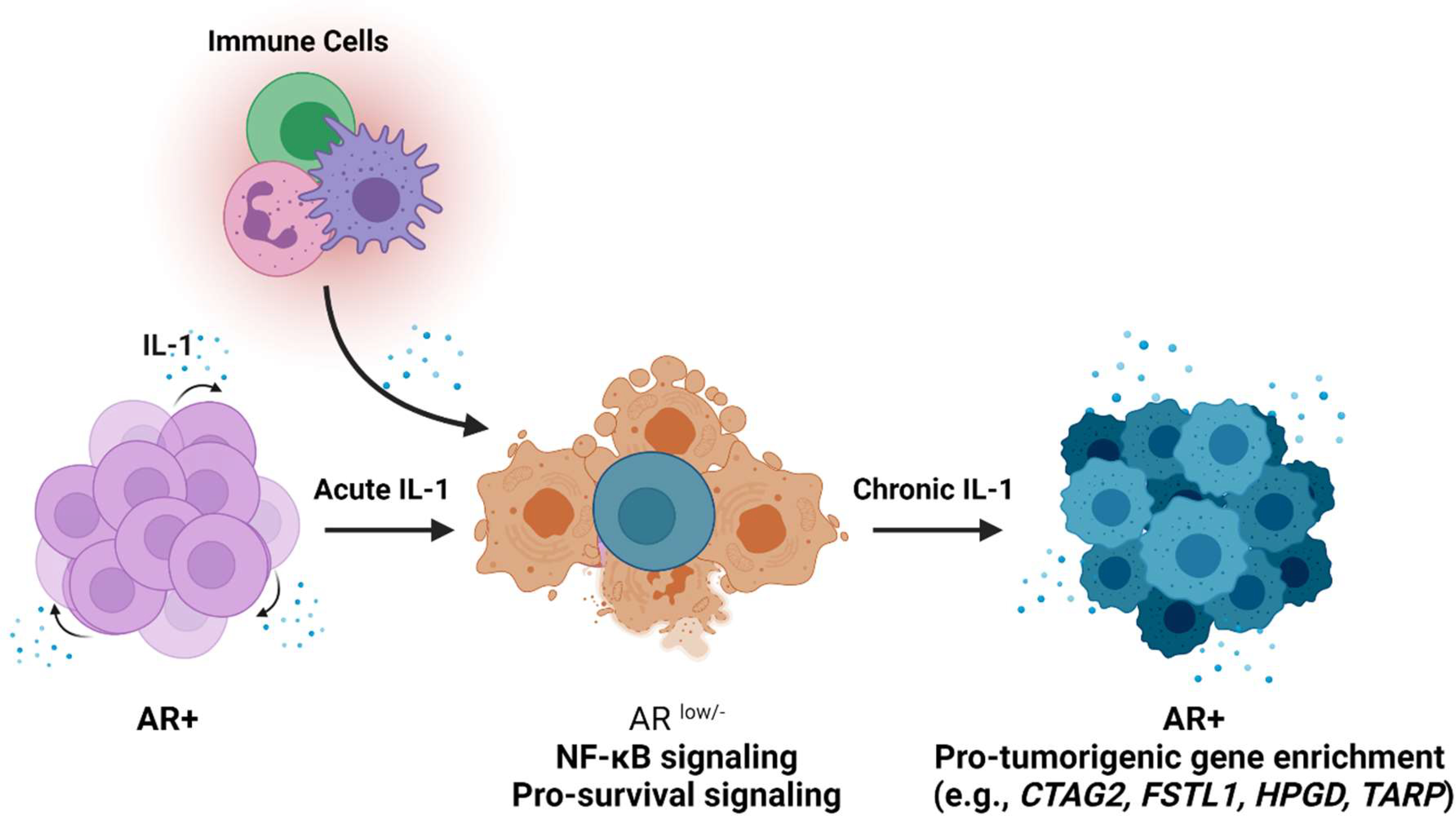
The effects of acute and chronic IL-1 signaling on AR^+^ PCa cells. Acute IL-1 signaling from tumor cell autocrine signaling and immune cell paracrine signaling causes AR-dependent PCa cells to lose AR expression and activity, leading to cell death. The surviving PCa cell subpopulation remains AR^low/−^, shows canonical IL-1-NF-kB signaling, and elicits pro-survival proteins and pathways. If acute inflammation is not resolved, chronic IL-1-exposed PCa cells evolve insensitivity to exogenous IL-1-induced signaling, thereby regaining AR expression and activity and acquiring pro-survival pathways that contribute to tumor progression. (Created with BioRender.com).

Taken together, we propose the following model (Figure 5) wherein acute IL-1 exposure is anti-tumorigenic, reducing cell viability and proliferation and repressing AR and AR activity in androgen dependent PCa cells. If the acute inflammation is left unresolved, the PCa cell subpopulation that survives the initial acute IL-1 assault evolves insensitivity to chronic IL-1 exposure and emerges more fit to contribute to PCa tumor growth and progression. It remains unknow if the conserved loss of sensitivity to chronic IL-1 exposure among PCa cell lines is due to the same or different intracellular mechanism; but the culmination of epigenetic and gene expression changes that result from chronic IL-1 exposure appear to diverge among PCa cell lines and likely reflects the inherent genomic difference among cell lines. To this end, while AR and AR activity are restored in both LNCaP [16] and MDA-PCa-2b chronic IL-1 sublines (Figure 1), subsequent AR dependency and therapeutic response may differ based on the differing epigenetic and gene expression landscapes that evolve. Thus, we can take advantage of our current cell line models and expand them to patient derived tumor models to understand how chronic IL-1 inflammation can affect PCa disease and treatment in individuals.

## Supplementary Material

JCS-20-058_Supplementary file

## Data Availability

The data and generated cell lines that support the findings of this study are available on request from the corresponding author. RNA sequencing data is publicly available at GEO NCBI, accession GSE181229.
