# Supplementary material for: Chronic IL-1 Exposed AR+ PCa Cell Lines Show Conserved Loss of IL-1 Sensitivity and Evolve Both Conserved and Unique Differential Gene Expression Profiles
Source: J Cell Signal. Author manuscript; Available in PMC 2022 Jan 4. (PMC8725614)
Supplement: JCS-20-058_Supplementary file [file NIHMS1755243-supplement-JCS-20-058_Supplementary_file.zip › Supplementary Figures.pdf]

Supplementary Figures

A

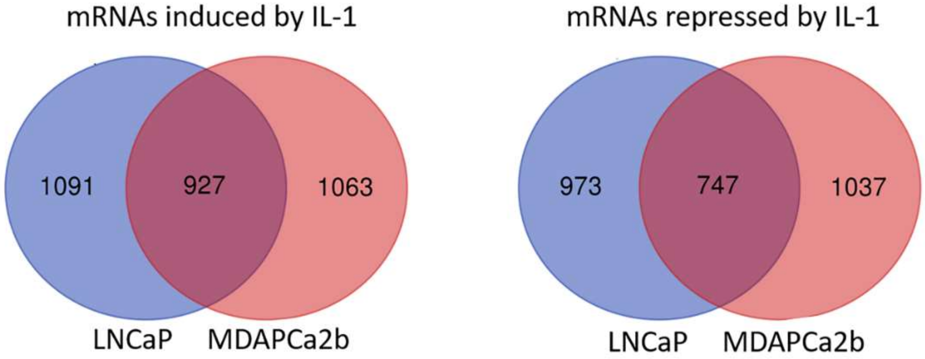

B

GO GENE SET	BIOLOGICAL PROCESS	ENRICHMENT RATIO	pValue
UPREGULATED			
GO:0034097	response to cytokine	2.38	0
GO:0006952	defense response	2.19	0
GO:0019221	cytokine-mediated signaling pathway	2.68	2.22E-16
GO:0071345	cellular response to cytokine stimulus	2.31	6.66E-16
GO:0006955	immune response	1.88	6.66E-16
GO:0045087	innate immune response	2.45	2.66E-15
GO:0051707	response to other organism	2.26	3.15E-13
GO:0043207	response to external biotic stimulus	2.25	3.58E-13
GO:0052696	flavonoid glucuronidation	22.74	5.88E-13
GO:0009607	response to biotic stimulus	2.21	7.43E-13
GO:1902531	regulation of intracellular signal transduction	1.78	2.11E-12
GO:0006954	inflammatory response	2.35	5.76E-12
GO:0043062	extracellular structure organization	2.90	7.28E-12
GO:0007249	I-kappaB kinase/NF-kappaB signaling	3.42	1.51E-11
GO:0030198	extracellular matrix organization	3.01	2.10E-11
DOWNREGULATED			
GO:0042273	ribosomal large subunit biogenesis	11.74	0
GO:0070972	protein localization to endoplasmic reticulum	11.70	0
GO:0002181	cytoplasmic translation	9.31	0
GO:0006612	protein targeting to membrane	8.57	0
GO:0006402	mRNA catabolic process	6.37	0
GO:0006401	RNA catabolic process	6.24	0
GO:0042254	ribosome biogenesis	6.04	0
GO:0090150	establishment of protein localization to membrane	5.65	0
GO:0016072	rRNA metabolic process	5.50	0
GO:0006412	translation	5.28	0
GO:0006260	DNA replication	5.26	0
GO:0043043	peptide biosynthetic process	5.13	0
GO:0022613	ribonucleoprotein complex biogenesis	4.96	0
GO:0007059	chromosome segregation	4.96	0
GO:0022618	ribonucleoprotein complex assembly	4.91	0

Supplementary Figure 1: Conserved biological processes induced by IL-1 in both LNCaP and MDA-PCa-2b cell lines. (A) Venn diagram shows overlap in the number of mRNAs induced (left Venn) or repressed (right Venn) by IL-1b in LNCaP cells and by IL-1a and IL-1b in MDA-PCa-2b cells. Venn diagram gene lists are in Supplementary Table 1, Tab 3. The Venn diagram was created using http://bioinformatics.psb.ugent.be/webtools/Venn/. (B) The table lists Gene Ontology (GO) Biological Processes as determined by Over Representation Analysis using

WebGestalt functional enrichment analysis web tool. Listed are the top 15 biological processes upregulated or downregulated by IL-1 treatment in both LNCaP and MDA-PCa-2b cells. A more complete list is in Supplementary Table 1, Tab 4 (upregulated biological processes) and Tab 5 (downregulated biological processes).

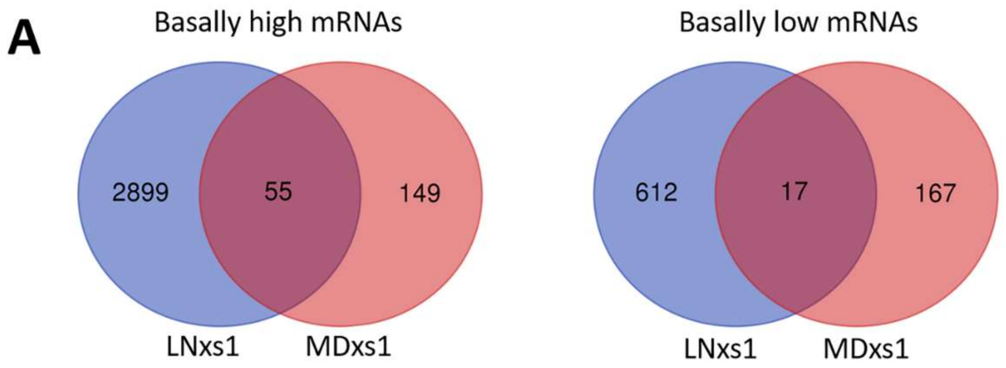

B

GO GENE SET	BIOLOGICAL PROCESS	ENRICHMENT RATIO	pValue
UPREGULATED			
GO:0052696	flavonoid glucuronidation	22.74	5.88E-13
GO:0052697	xenobiotic glucuronidation	18.60	2.98E-11
GO:0009812	flavonoid metabolic process	13.64	2.31E-09
GO:0052695	cellular glucuronidation	10.77	3.64E-08
GO:0034340	response to type I interferon	5.62	2.58E-11
GO:0060337	type I interferon signaling pathway	5.41	4.28E-10
GO:0071357	cellular response to type I interferon	5.41	4.28E-10
GO:0060333	interferon-gamma-mediated signaling pathway	4.76	2.83E-08
GO:0048525	negative regulation of viral process	4.65	4.13E-08
GO:0002526	acute inflammatory response	3.69	1.49E-08
GO:0007156	homophilic cell adhesion via plasma membrane adhesion molecules	3.65	4.98E-09
GO:0060759	regulation of response to cytokine stimulus	3.63	1.13E-08
GO:0043122	regulation of I-kappaB kinase/NF-kappaB signaling	3.51	8.65E-11
GO:0007249	I-kappaB kinase/NF-kappaB signaling	3.42	1.51E-11
GO:0034341	response to interferon-gamma	3.32	2.34E-08
DOWNREGULATED			
GO:0009266	response to temperature stimulus	23.69	1.86982E-05
GO:0034605	cellular response to heat	36.44	6.84712E-05
GO:0009628	response to abiotic stimulus	6.36	0.000173268
GO:0009408	response to heat	25.69	0.000193206
GO:0051085	chaperone cofactor-dependent protein refolding	88.18	0.000227263
GO:0051084	'de novo' posttranslational protein folding	74.40	0.000320377
GO:0006458	'de novo' protein folding	66.13	0.00040615
GO:1900034	regulation of cellular response to heat	54.11	0.000607533
GO:0032781	positive regulation of ATPase activity	44.09	0.000914606
GO:0061077	chaperone-mediated protein folding	42.52	0.000983328
GO:0043462	regulation of ATPase activity	30.52	0.00189736
GO:0001661	conditioned taste aversion	238.09	0.00419362
GO:2000609	regulation of thyroid hormone generation	238.09	0.00419362
GO:0010286	heat acclimation	238.09	0.00419362
GO:0070370	cellular heat acclimation	238.09	0.00419362

Supplementary Figure 2: Conserved upregulated or downregulated biological processes in both LNCaP and MDA-PCa-2b chronic IL-1 sublines. (A) Venn diagram shows overlap in the number of mRNAs basally high (left Venn) or basally low (right Venn) in LNas1, LNbs1, MDA-as1, and MDA-bs1 chronic IL-1 sublines (LNxs1 (LNas1,

LNbs1), MDxs1 (MDA-as1, MDA-bs1)). Venn diagram gene lists are in Supplementary Table 1, Tab 12. The Venn diagram was created using http://bioinformatics.psb.ugent.be/webtools/Venn/. (B) The table lists Gene Ontology (GO) Biological Processes as determined by Over Representation Analysis using WebGestalt functional enrichment analysis web tool. Listed are the top 15 biological processes basally high (upregulated) or basally low (downregulated) cells. A more complete list is in Supplementary Table 1, Tab 13 (upregulated biological processes) and Tab 14 (downregulated biological processes).
